# Design of ternary additive for organic photovoltaics: a cautionary tale†

**DOI:** 10.1039/d2ra00540a

**Published:** 2022-03-30

**Authors:** Chithiravel Sundaresan, Pierre Josse, Mário C. Vebber, Jaclyn Brusso, Jianping Lu, Ye Tao, Salima Alem, Benoît H. Lessard

**Affiliations:** Department of Chemical & Biological Engineering, University of Ottawa 161 Louis Pasteur Ottawa ON K1N 6N5 Canada benoit.lessard@uottawa.ca; Advanced Electronics and Photonics Research Centre, National Research Council of Canada Ottawa ON K1A 0R6 Canada salima.alem@nrc-cnrc.gc.ca; Department of Chemistry and Biomolecular Science, University of Ottawa 150 Louis-Pasteur Pvt Ottawa ON K1N 6N5 Canada; School of Electrical Engineering and Computer Science, University of Ottawa 800 King Edward Ave. Ottawa ON K1N 6N5 Canada

## Abstract

Silicon phthalocyanines as ternary additives are a promising way to increase the performance of organic photovoltaics. The miscibility of the additive and the donor polymer plays a significant role in the enhancement of the device performance, therefore, ternary additives can be designed to better interact with the conjugated polymer. We synthesized *N*-9′-heptadecanyl-2,7-carbazole functionalized SiPc ((CBzPho)_2_-SiPc), a ternary additive with increased miscibility in poly[*N*-90-heptadecanyl-2,7-carbazole-alt-5,5-(4′,7′-di-2-thienyl-2′,1′,3′-benzothiadiazole)] (PCDTBT). The resulting additive was included into PCDTBT and [6,6]-phenyl C_71_ butyric acid methyl ester as bulk (PC_71_BM) heterojunction OPV devices as a ternary additive. While the (CBzPho)_2_-SiPc demonstrated strong EQE >30% contribution in the range of 650–730 nm, the overall performance was reduced because (CBzPho)_2_-SiPc acted as a hole trap due to its high-lying HOMO energy level. This study demonstrates the importance of the solubility, miscibility, and energy level engineering of the ternary additive when designing organic photovoltaic devices.

## Abstract 2

Silicon phthalocyanines with carbazole axial functional groups were synthesized to improve the miscibility in PCDTBT and for use as ternary additives in organic photovoltaics.

## Graphical abstract

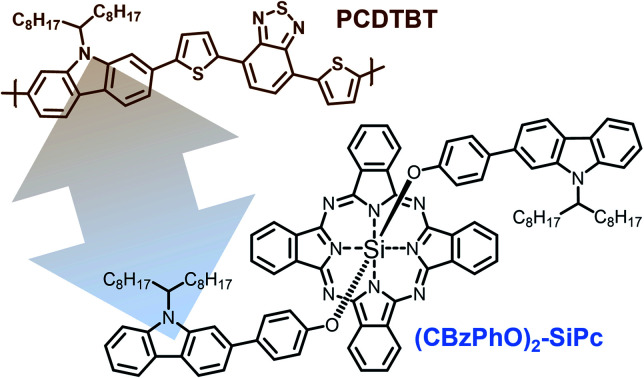

## Introduction

**Fig. 1:**
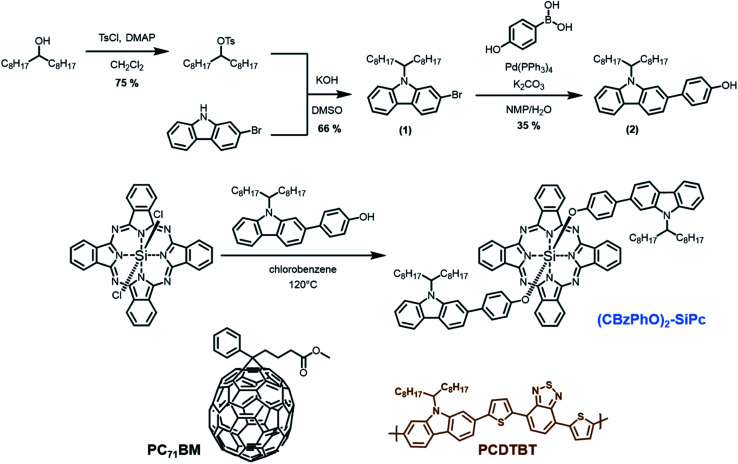
Synthesis of bis(*N*-9′-heptadecanyl-2,7-carbazole-phenoxy) silicon phthalocyanine ((CBzPhO)_2_-SiPc) in addition to the chemical structure of PCDTBT and PC_71_BM.

Organic photovoltaics (OPVs) are proving to be an exciting, flexible, semitransparent light harvesting technology with the potential to reduce power requirements and provide clean energy. The typical OPV requires complimentary donor/acceptor semiconductors to harvest photons and convert them into current. A strategy to improve device performance is to use functional ternary additives, which can be added to existing donor–acceptor systems while providing additional functionality such as increased stability or increased photogeneration.^1–4^ Silicon phthalocyanines (SiPcs) are a promising class of ternary additive due to their low manufacturing cost, industrial abundance and ability to efficiently transport electrons.^5–7^ Bis(tri-hexylsilyl) SiPcs ((3HS)_2_-SiPc) and bis(tri-butylsilyl) SiPcs ((3BS)_2_-SiPc) were previously employed as a ternary additive in poly(3-hexylthiophene):phenyl-C_61_-butyric acid methyl ester (P3HT:PC_61_BM) bulk heterojunction (BHJ) OPV devices and provided a 20% increase in power conversion efficiency due to the additional absorption at 685 nm^8,9^ Following these initial studies, researchers have explored engineering new SiPcs through increasing the conjugation of the axial groups with pyrenes to improve absorption in the UV region^10,11^ as well as increasing the conjugation of the macrocycle to increase absorption in the NIR region.^12–14^ SiPcs have even been modified to impart both additional photogeneration and increased device stability through active layer crosslinking.^15^ Our group^16^ and others^14,17^ have also found that the length of the axial groups and the resulting change in solubility play a critical role in the effectiveness of the ternary additive in a P3HT:PC_61_BM system. Poly[*N*-9′-heptadecanyl-2,7-carbazole-alt-5,5-(4′,7′-di-2-thienyl-2′,1′,3′-benzothiadiazole)] (PCDTBT, Fig. 1), is another conjugated polymer of interest due to its high potential for indoor lighting applications, it's chemical scale-up potential and its ability to be easily printed on large areas.^18,19^ Recently, our groups reported (3HS)_2_-SiPc to also be an effective ternary additive in PCDTBT:PC_71_BM BHJ devices, leading to an increased photogeneration efficiency compared to the baseline devices without additive. In this study we reported that the choice of processing technique such as blade coating *versus* spin coating led to different optimal (3HS)_2_-SiPc loadings; further suggesting the molecular structure of the ternary additive and the film formation process are critical for improving the performance of the device.^20^ However, increased miscibility between donor polymer and ternary additive could lead to better phase separation between domains, which would improve exciton dissociation, charge transport and potentially enhance overall device performance.^21–24^ (3HS)_2_-SiPc has been characterized to be preferentially located at the interface of P3HT/PC_61_BM rather than the bulk phases, leading to favourable device performance due to enhanced charge and energy transfer between (3HS)_2_-SiPc and P3HT/PC_61_BM interfaces.^17^ However, when using PCDTBT:PC_71_BM the locations of SiPc based additives in the corresponding ternary thin films yet to be explored.

We believed that improving miscibility between the SiPc ternary additive and the PCDTBT domain would potentially improve the device performance. Therefore, in this study we designed and synthesized a *N*-9′-heptadecanyl-2,7-carbazole functional SiPc (Fig. 1), which contains the same functional group found in PCDTBT as a repeat unit. By matching the donor polymer with the axial groups of the additive, we surmise improved miscibility will improve the device performance. While the miscibility increased through surface energy calculations, the device performance did not. Cyclic voltammetry measurement showed that the HOMO energy level of the new additive is around −5.1 eV, which is located above PCDTBT (−5.4 eV). As a result, the new additive could function as hole traps in the devices.

## Experimental section

### Materials

PCDTBT (*M*_n_ = 36 kDa and *M*_w_ = 110 kDa) and PC_71_BM (>99%) were purchased from PCAS Canada Inc. and Nano-C, respectively, and used without any further purification. ZnO nanoparticles were prepared according to previous reports.^25^ Molybdenum oxide (MoO_3_) (>99.5%) was purchased from Sigma-Aldrich and used with no further purification. The carbazole SiPc based derivative was synthesized according to Fig. 1.

### Synthesis of bromo-*N*-9′-heptadecanyl-2,7-carbazole (1)

The following procedure was adapted from previously reported synthesis.^26^*p*-Toluenesulfonyl chloride (1.49 g; 7.80 mmol), heptadecan-9-ol (2.00 g, 7.80 mmol) and 4-dimethylaminopyridine (DMAP, 1.43 g; 11.70 mmol) were placed in a flame-dried 100 ml flask under N_2_ to which 40 ml of anhydrous CH_2_Cl_2_ was added. After stirring the reaction mixture overnight at room temperature, it was poured onto water and washed three times. The organic phase was separated, dried over MgSO_4_, then concentrated *in vacuo*. The resulting residue was purified by silica-gel column chromatography (90% hexanes/10% ethyl acetate as eluent) to give 2.4 g (75%) of a colorless oil. The oil was redissolved in 15 ml DMSO and added dropwise to a flame-dried round bottom flask under N_2_ containing bromocarbazole (500 mg; 2.03 mmol) and KOH (570 mg; 10.16 mmol) in 10 ml DMSO. After stirring overnight at room temperature, water (≈100 ml) was added and the reaction mixture was extracted with hexanes three times. The organic phase was separated, then washed with water before being dried over MgSO_4_ and concentrated by rotary evaporation. The resulting residue was purified by silica gel column chromatography using hexanes as eluent providing 667 mg (66%) of a colorless oil. ^1^H NMR (*δ*, CDCl_3_, RT, 300 MHz): 8.10 (m, 1H), 7.98 (m, 1H), 7.73 (m, 1H), 7.58 (m, 1H), 7.45 (m, 1H), 7.36 (m, 1H), 7.29 (m, 1H), 4.51 (m, 1H), 2.27 (m, 2H), 1.95 (m, 2H), 1.17 (m, 24H), 0.86 (t, 6H).

### Synthesis of phenoxy-*N*-9′-heptadecanyl-2,7-carbazole (2)

Boronic acid (250 mg; 1.82 mmol), Pd(PPh_3_)_4_ (19 mg; 16.51 mmol) and K_2_CO_3_ (342 mg; 2.48 mmol) were placed into a 250 ml Schlenk flask and degassed under vacuum. (1) (100 mg; 0.204 mmol) and solvents (NMP : H_2_O; 9 : 1 v/v; 100 ml) were placed into a separate flask and degassed by nitrogen bubbling. After 30 min, the liquid phase was transferred to the Schlenk flask *via* cannula. The reaction mixture was then immersed into an oil bath and stirred at 90 °C overnight under a nitrogen atmosphere. After cooling to room temperature, the reaction mixture was poured into water (≈400 ml) and stirred at room temperature for 30 min. The aqueous phase was extracted three times with hexanes. The organic phase was then washed with water, dried over MgSO_4_ and the solvent was removed by rotary evaporation. The obtained residue was purified by silica gel column chromatography using DCM as eluent leading to 290 mg (35%) of a colorless oil. ^1^H NMR (*δ*, CDCl_3_, RT, 300 MHz): 8.11 (dd, 2H), 7.68 (m, 1H), 7.61 (m, 1H), 7.58 (m, 1H), 7.51 (m, 1H), 7.38–7.41 (m, 2H), 7.22 (m, 1H), 6.96 (dt, 2H), 4.60 (m, 1H), 2.31 (m, 2H), 1.94 (m, 2H), 1.12–1.26 (m, 24H), 0.83–0.79 (t, 6H).

### Synthesis of bis(*N*-9′-heptadecanyl-2,7-carbazole-phenoxy) silicon phthalocyanine ((CBzPhO)_2_-SiPc)

Dichloro silicon phthalocyanine (Cl_2_-SiPc) was prepared according to previous reports.^27^ Cl_2_-SiPc (120 mg; 196 mmol) and (2) (290 mg; 588 mmol) were added to a 100 ml RBF with 30 ml chlorobenzene and the reaction mixture was stirred at 130 °C for 2 days under a nitrogen atmosphere. After cooling to room temperature, the solvent was removed by rotary evaporation. The crude material was purified by silica gel column chromatography using DCM as eluent affording 150 mg (40%) of a dark blue powder. ^1^H NMR (*δ*, CDCl_3_, RT, 400 MHz): 9.65–9.67 (m, 8H), 8.35–8.37 (m, 8H), 7.73–7.94 (ddd, 4H), 7.28–7.45 (m, 4H), 7.09–7.12 (m, 2H), 6.66–6.83 (m, 2H), 6.56–6.58 (m, 2H), 5.91–5.94 (m, 4H), 4.23–4.37 (m, 2H), 2.55 (dt, 4H), 1.70–2.11 (m, 8H), 1.03–1.14 (m, 48H), 0.75–0.84 (m, 12H).

### Materials characterization

UV-Vis absorption spectra of (CBzPho)_2_-SiPc in solution and ternary films and the total absorption spectra of OPV devices were measured using a PerkinElmer LAMBDA 950 UV/Vis/NIR spectrophotometer. The PCDTBT:PC_71_BM binary solution was prepared in 1,2-dichlorobenzene (*o*-DCB, HPLC grade) with weight ratio of 1 : 3 and a total concentration of 16 mg ml^−1^. The solution was stirred at 100 °C for 24 h. Ternary solutions were prepared by adding (CBzPho)_2_-SiPc into the binary solution with various weight contents ranging from 3 to 20 wt% and stirred for an additional 24 h at 70 °C.

Cyclic voltammograms (CV) were obtained using a VersaSTAT 3 potentiostat, a polished platinum disk as the working electrode, a coiled platinum wire as the counter electrode, and a Ag/AgCl electrode as the reference.^16^ These experiments were carried out in dichloromethane (DCM) solutions with tetrabutylammonium perchlorate as the supporting electrolyte. The highest occupied molecular orbital (HOMO) energy levels were estimated according to the empirical correlation *E*_HOMO_ (eV) = −(*E*_ox, onset_ − *E*_ox Fc/Fc^+^, onset_) − 4.80 eV, where *E*_ox, onset_ and *E*_ox Fc/Fc^+^, onset_ are the onset oxidation potentials of the sample and the ferrocene standard, respectively.^16^ Lowest occupied molecular orbital (LUMO) levels were estimated from combining HOMO from CV with onset of the UV/Vis absorption spectra (either solution or solid phase). Tapping-mode atomic force microscopy (AFM) images were acquired with a Veeco scanning probe microscope using a Nanodrive controller with MikroMasch NSC-15 AFM tips and resonant frequencies of ∼300 kHz. Water contact angle was measured on a spin-coated film of corresponding material by Folio instruments using pure water. The surface energy of all the pristine and ternary films were estimated from the contact angle.

### Device fabrication

The blade-coated devices were fabricated on flexible ITO-coated polyethyleneterapthalate (PET) sheets with dimension of 12 × 15 cm^2^, purchased from Sigma Aldrich. The PET sheet thickness, sheet resistance and thickness of the ITO are 125 μm, 60 Ω sq^−1^ and 130 nm, respectively. The ITO sheets were patterned using a screen printable etching paste (SolarEtch AXS Type 20). The etching paste was printed using an EKRA X1-SL flatbed screen printer with a 350-mesh stainless steel screen. After curing the paste at 120 °C for 10 min, the underlying ITO was etched, and then the paste was removed with deionized water. The patterned ITO sheets were afterward scrubbed with a detergent solution and then rinsed with DI water, followed by an additional 5 min sonication in acetone (purity 99.5%), and isopropyl alcohol (IPA, purity 99.999%) sequentially. The ITO sheets were then blown dry with a nitrogen air gun. The cleaned ITO/PET sheets were treated with an oxygen plasma for 30 s. A thin layer of ZnO (∼15 nm) was blade coated at a speed of 2.5 mm s^−1^ with a blade gap of 0.3 mm by dropping 250 μl of ZnO nanoparticle solution at the beginning of substrate area. The resulting films were then dried on a hot plate at 110 °C for 10 min in air. The binary baseline and ternary additive films were blade coated at a speed of 15 mm s^−1^ with a blade gap of 0.3 mm by dropping 150 μl of blended solutions. The temperature of the blade stage was set at 40 °C during the deposition process, resulting in a film thickness of 75–80 nm. The blade coating of ZnO and active layers was carried out in air under laminar flow hood. The OPV device structure was completed by vacuum deposition (base pressure ∼2 × 10^−7^ Torr) of 10 nm of MoO_*x*_ and 100 nm of silver (Ag). All the OPV devices have an active area of 1 cm^2^. The thickness of films was measured by a Dektak profilometer and a ZYGO 45 NewView 7300 optical profiler.

### Electrical characterization

All the OPV device characterizations were performed in ambient air. The photovoltaic parameters were extracted from the current density–voltage (*J*–*V*) characteristics measured using a Keithley 2400 digital source meter under AM 1.5G irradiation of 100 mW cm^−2^ (Science Tech SS 500 W solar simulator). A calibrated Si photodiode with a KG-5 filter, purchased from PV measurements Inc, was used to adjust the light intensity of solar simulator. The external quantum efficiency (EQE) spectrum was measured using a Jobin-Yvon Triax spectrometer, a Jobin-Yvon xenon light source, a Merlin lock-in amplifier, a calibrated Si UV detector, and an SR570 low noise current amplifier. The short-circuit current density (*J*_SC_) of all the blade-coated devices reported in this study were verified from the wavelength integration of the product of the EQE curve and the standard AM 1.5G solar spectrum.

## Results and discussion

### Synthesis and characterization of (CBzPhO)_2_-SiPc

**Fig. 2:**
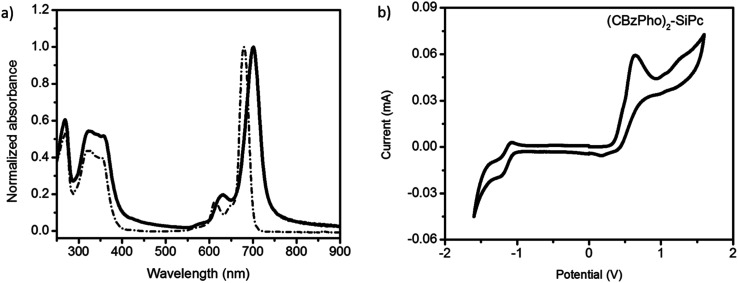
(a) UV-Vis spectra of (CBzPho)_2_-SiPc in a chloroform solution (dashed line) and thin film (solid line), (b) redox scans of cyclic voltammograms for (CBzPho)_2_-SiPc.

(CBzPhO)_2_-SiPc was synthesized by phenoxylation of Cl_2_-SiPc through standard conditions (Fig. 1).^28^ The custom phonoxy derivative was designed to mimic the solubilizing carbazole repeat unit of PCDTBT with the intention to increase miscibility with the donor phase in the resulting BHJ OPVs. A yield of 40% was obtained for the phenoxylation, which is typical for coupling reactions of Cl_2_-SiPc.^28,29^ the chemical structure was verified by ^1^H NMR and electrospray ionization (ESI) mass spectra (Fig. S1–S4†). The (CBzPhO)_2_-SiPc was characterized by UV-Vis absorption spectroscopy revealing a characteristic peak at 714 nm in chloroform solution and 720 nm in a neat thin film (Fig. 2a). These values correspond to the Q-band of the silicon phthalocyanine core, which is slightly red shifted by 42–45 nm compared to typical silyl functional SiPcs with a *λ*_max_ = 669 to 672 nm.^30^ The UV-Vis spectra of (CBzPhO)_2_-SiPc also present an additional broad peak between 300 and 400 nm (Fig. 2a), which is typically not present in silyl functional SiPcs and is likely due to the carbazole axial groups. A similar hump was observed in pyrene functional SiPc derivatives.^10^ The HOMO–LUMO or energy band gap (*E*_GAP_) for (CBzPhO)_2_-SiPc was estimated from the onset of the UV-Vis spectra and found to be equal to 1.73 eV and 1.85 eV from solution and thin film, respectively, which is similar to previous reports of silyl functional SiPcs with *E*_GAP_ = 1.80–1.82 eV (solution) and *E*_GAP_ = 1.85 (film).^30^

Cyclic voltammetry was performed on (CBzPhO)_2_-SiPc in DCM solvent. The oxidation of the derivative was measured relative to Ag/AgCl reference electrode using a platinum working electrode.^16^ An oxidation of potential of *E*_ox_ = 0.36 V and a reduction potential of *E*_red_ = −0.96 V were obtained (Fig. 2b). The highest occupied molecular orbital energy level, *E*_HOMO_ = −5.1 eV was obtained for (CBzPhO)_2_-SiPc which is relatively shallow compared to typical silyl functionalized SiPcs with *E*_HOMO_ = −5.3 eV (Table S4†).^30^ The lowest unoccupied molecular orbital energy level, *E*_LUMO_, was calculated through the addition of *E*_HOMO_ and solid state *E*_GAP_ and found to be equal to −3.3 eV which is also relatively shallow compared to typical silyl functional SiPcs with *E*_LUMO_ = −3.5 eV.^30^

### Miscibility and film morphology

**Fig. 3:**
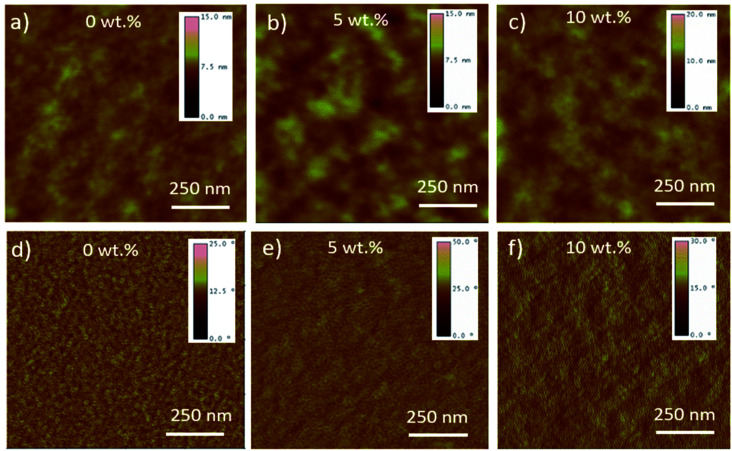
Tapping mode AFM height (above) and corresponding phase (below) images of PCDTBT:PC_71_BM:(CBzPho)_2_-SiPc ternary blends with various (CBzPho)_2_-SiPc contents deposited by blade-coating (a and d) 0 wt% (rms roughness = 0.71 nm); (b and e) 5 wt% (rms roughness = 0.91 nm); (c and f) 10 wt% (rms roughness = 2.5 nm).

We performed water contact angle measurements on neat films of PCDTBT, PC_71_BM, and (CBzPho)_2_-SiPc as well as blended ternary films with various weight contents of (CBzPho)_2_-SiPc to explore their relative hydrophobicity and draw insight on respective miscibility. The surface energy was estimated using the Neumanss's method.^31^Table 1 tabulates water contact angles and surface energy values of pristine thin films prepared on quartz substrates. It has been reported that additives with a surface energy value intermediate between that of the donor and acceptor materials will migrate to the interface^17,32,33^ As designed, the surface energy of (CBzPho)_2_-SiPc is almost identical to that of PCDTBT (∼24 N mm^−1^) where the contact angles of (CBzPho)_2_-SiPc and PCDTBT are 97° and 96°, respectively. As a comparison, the previously reported (3HS)_2_-SiPc has a contact angle of ∼101°.^32^ We performed the same measurements on ternary blends of PCDTBT, PC_71_BM, and (CBzPho)_2_-SiPc and found that the addition of (CBzPho)_2_-SiPc led to a slight increase in the contact angle from 93.2° for 3wt% to 95.3° for 20 wt% and a slight drop in the corresponding surface energy from 26.5 N mm^−1^ to 25.4 N mm^−1^ (Table 1 and Fig. S7†). The surface energy of (CBzPho)_2_-SiPc is decreased and almost matching with PCDTBT. This suggests an enhanced miscibility and a decreased likelihood of phase separation between (CBzPho)_2_-SiPc and PCDTBT. The atomic force microscopy (AFM) images (Fig. 3) show an increase in the feature size and root-mean square roughness (RMS) of ternary films as the (CBzPho)_2_-SiPc concentration increased from 0 to 10%. While an increased RMS of 2.5 nm was measured when 10 wt% of (CBzPho)_2_-SiPc is added, no large islands are present in the phase images, suggesting the SiPc additive is not phase separating into a distinct third phase.

**Table 1:** Surface characterization of thin films

Sample^a^	Contact angle (°)	Surface energy^c^ (N mm^−1^)	Ref.
PCDTBT	96.7 (±0.4)	24.3 (±0.3)	This work
PC_71_BM	85.5 (±0.5)	31.3 (±0.3)	This work
(CBzPho)2-SiPc	97.3 (±0.4)	23.6 (±0.4)	This work
(3HS)2-SiPc	100.8(±0.5)	21.8 (±0.4)	Ref. 32
3 wt% (CBzPho)2-SiPc^b^	93.2 (±0.3)	26.5 (±0.3)	This work
20 wt% (CBzPho)2-SiPc^b^	95.3 (±0.4)	25.4 (±0.3)	This work

a Pristine thin films spin casted on quartz substrates.

b Ternary thin films spin casted on quartz substrates.

c Surface energy was estimated using the Neumann's method.

### Organic photovoltaic device characterization

**Fig. 4:**
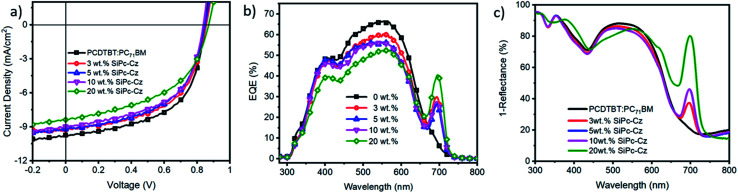
(a) *J*–*V* characteristics of PCDTBT:PC_71_BM:(CBzPho)_2_-SiPc ternary BHJ OPV devices fabricated by blade coating on ITO/PET substrates, (b) corresponding EQE curves and (c) UV-Vis total absorption spectra of OPV devices.

(CBzPho)_2_-SiPc was used as a ternary additive in BHJ PCDTBT:PC_71_BM inverted devices with the following structure: ITO-PET/ZnO/PCDTBT:PC_71_BM:(CBzPho)2-SiPc/MoO_*x*_/Ag. (CBzPho)_2_-SiPc was added at four different concentrations 3, 5, 10 and 20 wt% relative to PCDTBT:PC_71_BM binary blend. Fig. 4a shows the *J*–*V* characteristics of the OPV devices as a function of (CBzPho)_2_-SiPc concentrations, and the corresponding device parameters are summarized in Table S2.† The PCDTBT:PC_71_BM baseline device (0% of (CBzPho)_2_-SiPc) exhibited an average PCE of 4.5% with an open circuit voltage (*V*_OC_) of 0.84 V, a short-circuit current density (*J*_SC_) of 9.4 mA cm^−2^, a fill factor (FF) of 56.6%, which is consistent with previous reports.^20,34–36^ The addition of (CBzPho)_2_-SiPc compound as a ternary additive, resulted in a consistent decrease in overall device performance compared to the baseline devices. The addition of as little as 3 wt% of (CBzPho)_2_-SiPc led to a drop in *J*_SC_ and FF values. This reduction is likely due to energy offset between PCDTBT and (CBzPho)_2_-SiPc (Fig. S5†). Although (CBzPho)_2_-SiPc has shown better miscibility with PCDTBT, the cascade energy transfer at the interface of PCDTBT and (CBzPho)_2_-SiPc is no longer favorable. The devices exhibit significant charge recombination losses, as investigated by light intensity-dependant measurements (Fig. S8, S9, and Table S3†), which is in correlation with the coarse texture observed in the AFM images. This charge recombination loss has been previously associated to the SiPc additives not being present at the interface which is consistent with our contact angle measurements.^17^

External quantum efficiency (EQE) spectra show that the addition of the (CBzPho)_2_-SiPc compound significantly enhanced the photon conversion in the wavelength region of 680–730 nm, corresponding to the (CBzPho)_2_-SiPc peak absorption (Fig. S6†). However, the addition of (CBzPho)_2_-SiPc decreased the PCDTBT:PC_71_BM contribution in the region of 400–650 nm, which is consistent with the drop in *J*_SC_ likely due to the disruption of desired BHJ morphology from the segregation at donor phase.^8,37^ The total absorption spectra of the devices (Fig. 4c) show about the same pattern as the EQE spectra. At loadings of 3–10 wt% of (CBzPho)_2_-SiPc, we observe a small drop in absorbance between 450 nm and 600 nm, probably due to the thickness optimization (∼75 nm), but its drop is much smaller than the drop in the EQE spectra, which indicates that more excited excitons failed to contribute to the photocurrent with increasing (CBzPho)_2_-SiPc content, probably due to a hole trapping effect in the BHJ by (CBzPho)_2_-SiPc. When loading 20 wt% of (CBzPho)_2_-SiPc, we notice a shoulder peak appearing in the total absorption spectra at 650 nm, suggesting that (CBzPho)_2_-SiPc is disrupting the PCDTBT domain but remains blended in the PCDTBT domain unlike in previously reported P3HT systems using (3HS)_2_-SiPc,^8,38^ which led to a tendency to aggregate into a third unique SiPc domain at high loadings.

## Conclusion

We designed and synthesized an *N*-9′-heptadecanyl-2,7-carbazole functional SiPc ((CBzPho)_2_-SiPc) with matching functionality to PCDTBT to improve their miscibility. UV-Vis absorption spectroscopy and electrochemical characterization of (CBzPho)_2_-SiPc revealed slightly shallower HOMO and LUMO energy levels compared to typical silyl functionalized SiPcs. The thin film contact angle and the resulting surface energy calculations suggest very similar characteristics between (CBzPho)_2_-SiPc and PCDTBT suggesting a good miscibility. As a ternary additive in PCDTBT:PC_71_BM OPV devices, CBzPho_2_-SiPc showed a significant EQE contribution from 680 to 730 nm; however, decreased PCDTBT:PC_71_BM contribution, resulting in a net loss in the short-circuit current density suggested the additive was not at the interface between donor and acceptor and that the (CBzPho)_2_-SiPc is likely dispersed in the PCDTBT domain and acted as hole traps leading to a drop in the device performance or this drop in performance could simply be a result of poor ternary film morphology. This study further suggests the importance of the solubility and miscibility of the ternary additive when designing organic photovoltaic devices.

## Conflicts of interest

The authors have no conflicts to declare.

## Supplementary Material

RA-012-D2RA00540A-s001
