# Supplementary material for: Design of ternary additive for organic photovoltaics: a cautionary tale
Source: RSC Adv. 2022 Mar 30;12(16):10029–36. doi: 10.1039/d2ra00540a (PMC8965687; doi:10.1039/d2ra00540a)
Supplement: RA-012-D2RA00540A-s001 [file RA-012-D2RA00540A-s001.pdf]

Electronic Supplementary Material (ESI) for RSC Advances.
This journal is © The Royal Society of Chemistry 2022

Design of Ternary Additive for Organic Photovoltaics: A Cautionary Tail

Chithiravel Sundaresan,[1,2] Pierre Jossse[1,3], Mário C. Vebber[1], Jaclyn Brusso[3], Jianping Lu[2], Ye Tao[2], Salima Alem[2,], and Benoît H. Lessard[1,4*]*

[1]Department of Chemical & Biological Engineering, University of Ottawa, 161 Louis Pasteur, Ottawa, ON, Canada, K1N 6N5
[2]Advanced Electronics and Photonics Research Centre, National Research Council of Canada, Ottawa, ON, K1A 0R6, Canada
[3]Department of Chemistry and Biomolecular Science , University of Ottawa, 150 Louis-Pasteur Pvt, Ottawa, ON, Canada, K1N 6N5
[4]School of Electrical Engineering and Computer Science, University of Ottawa, 800 King Edward Ave. Ottawa, ON, Canada, K1N 6N5

*corresponding authors: benoit.lessard@uottawa.ca (B.H.L) and salima.alem@nrc-cnrc.gc.ca (S.A.)

Electronic supporting Information

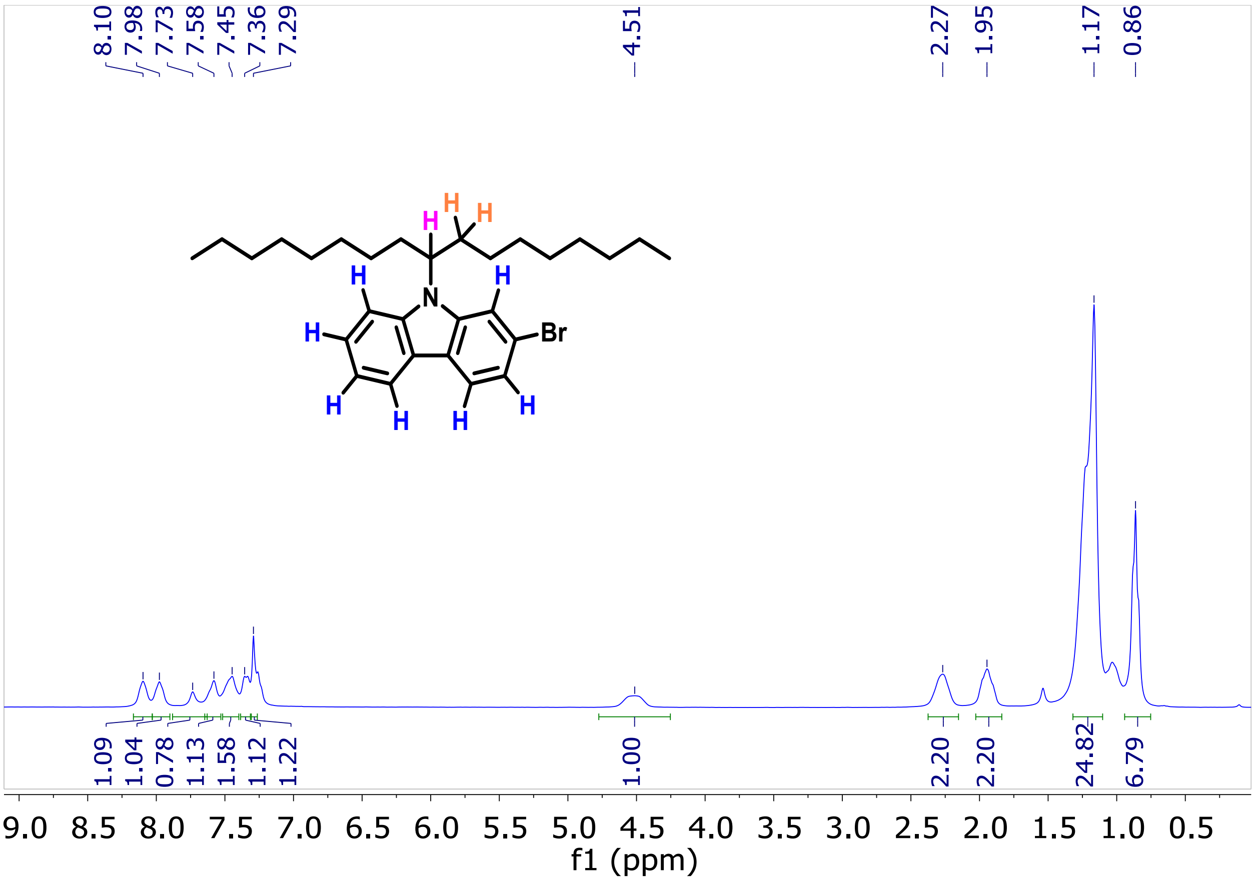

Figure S1. 1H NMR spectra recorded at 300K in CDCl3

^{1}H NMR (δ, $CDCl_3$, RT, 300 MHz): 8.10 (m, 1H), 7.98 (m, 1H), 7.73 (m, 1H), 7.58 (m, 1H), 7.45 (m, 1H), 7.36 (m, 1H), 7.29 (m, 1H), 4.51 (m, 1H), 2.27 (m, 2H), 1.95(m, 2H), 1.17(m, 24H), 0.86 (t, 6H).

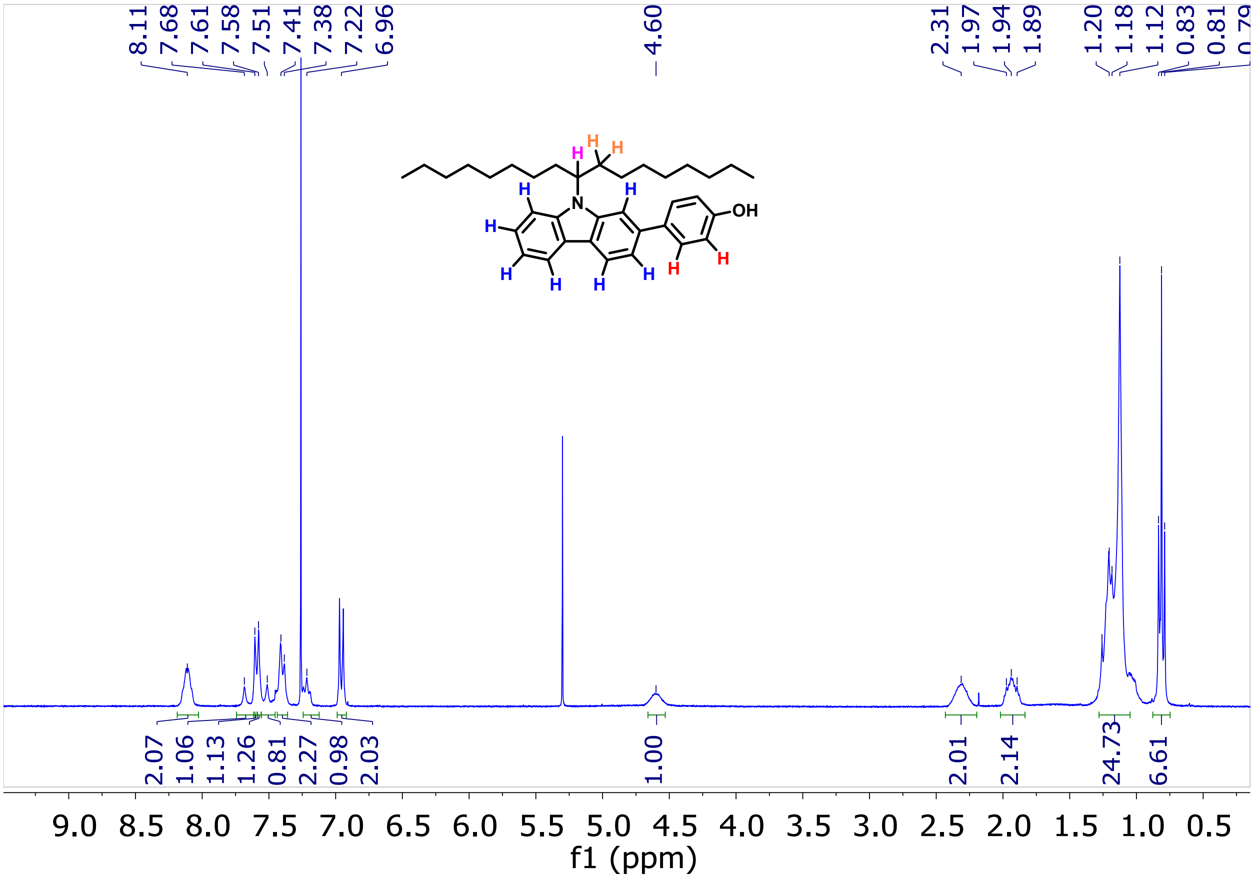

Figure S2. 1H NMR spectra recorded at 300K in CDCl3

^{1}H NMR (δ, $CDCl_3$, RT, 300 MHz): 8.11 (dd, 2H), 7.68 (m, 1H), 7.61 (m, 1H), 7.58 (m, 1H), 7.51 (m, 1H), 7.38−7.41 (m, 2H), 7.22 (m, 1H), 6.96 (dt, 2H), 4.60 (m, 1H), 2.31 (m, 2H), 1.94 (m, 2H), 1.12−1.26 (m, 24H), 0.83−0.79 (t, 6H).

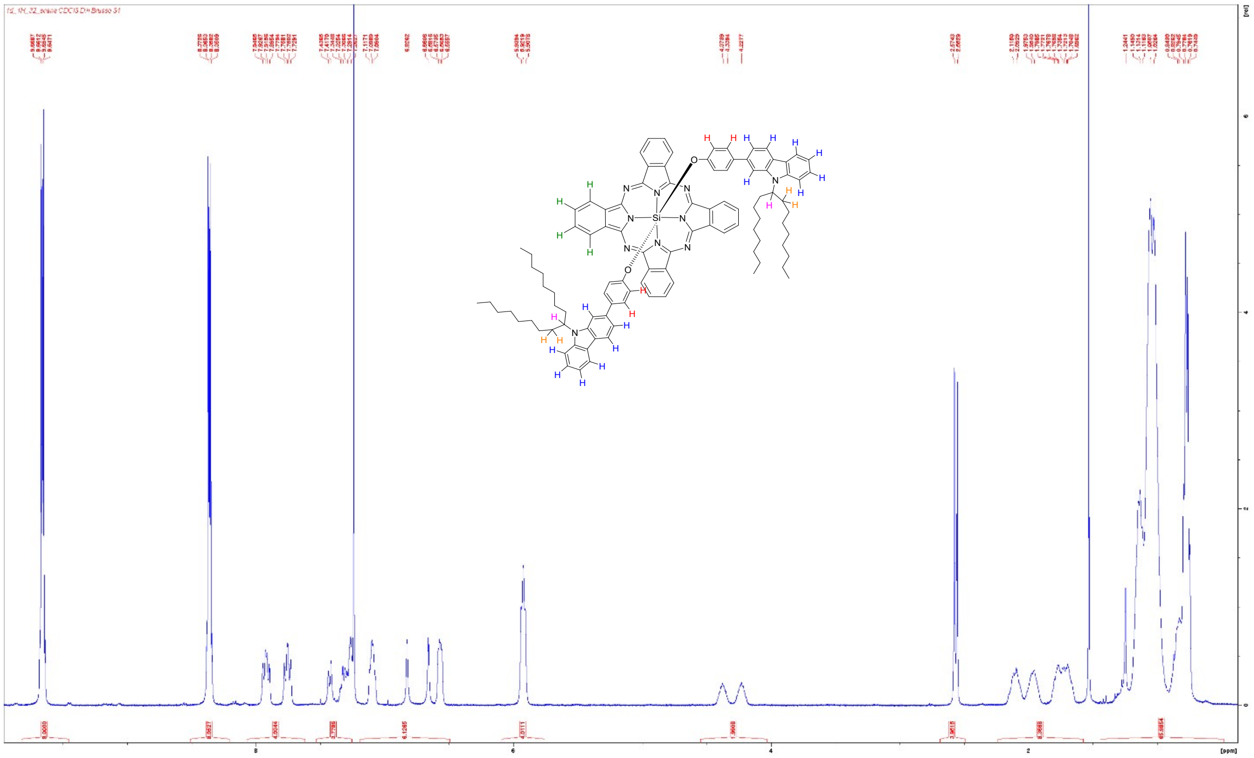

Figure S3. 1H NMR spectra recorded at 300K in CDCl3

^{1}H NMR (δ, $CDCl_3$, RT, 400 MHz): 9.65−9.67 (m, 8H), 8.35−8.37 (m, 8H), 7.73−7.94 (ddd, 4H), 7.28−7.45 (m, 4H), 7.09−7.12 (m, 2H), 6.66−6.83 (m, 2H), 6.56−6.58 (m, 2H), 5.91−5.94 (m, 4H), 4.23−4.37 (m, 2H), 2.55 (dt, 4H), 1.70−2.11 (m, 8H), 1.03−1.14 (m, 48H), 0.75−0.84 (m, 12H).

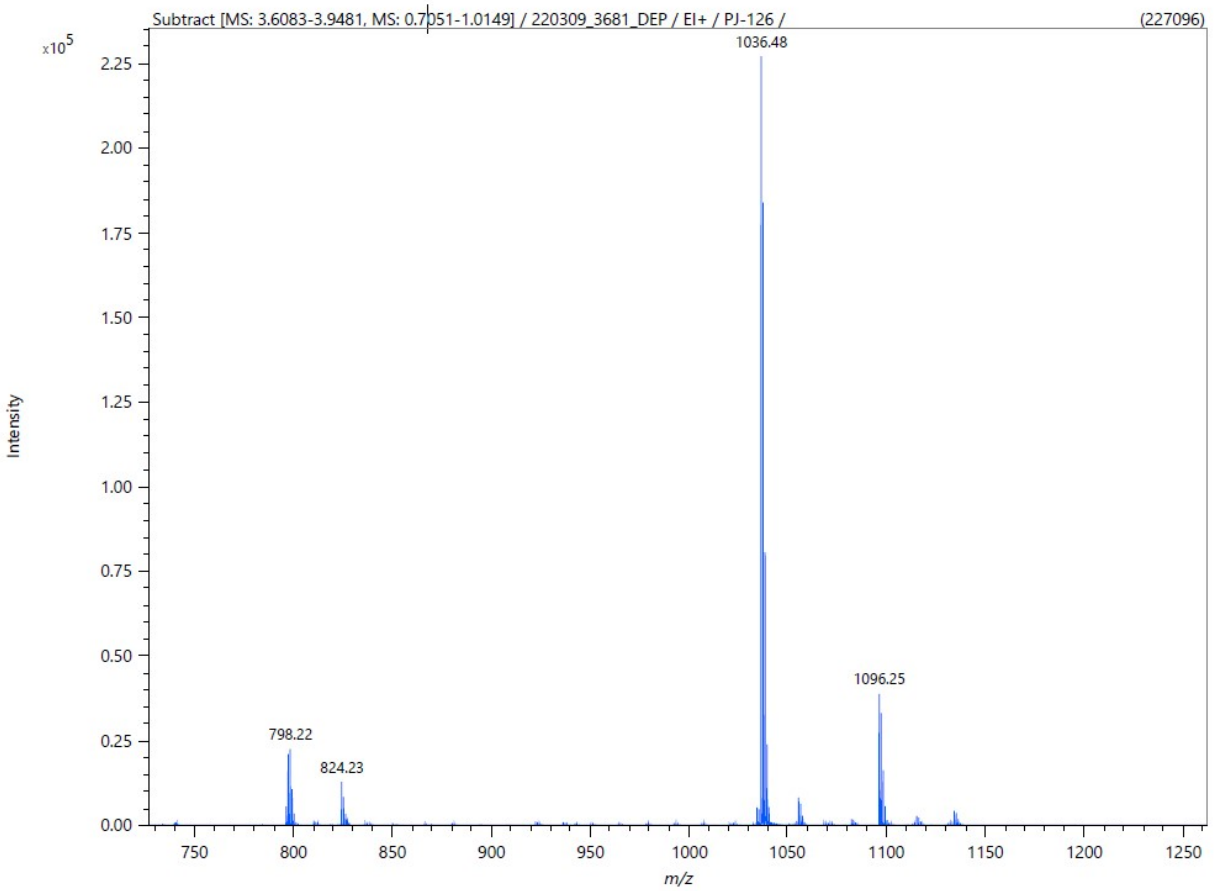

Figure S4. Electrospray ionization (ESI) Mass spectra plot of $(CBzPho)_2$-SiPc

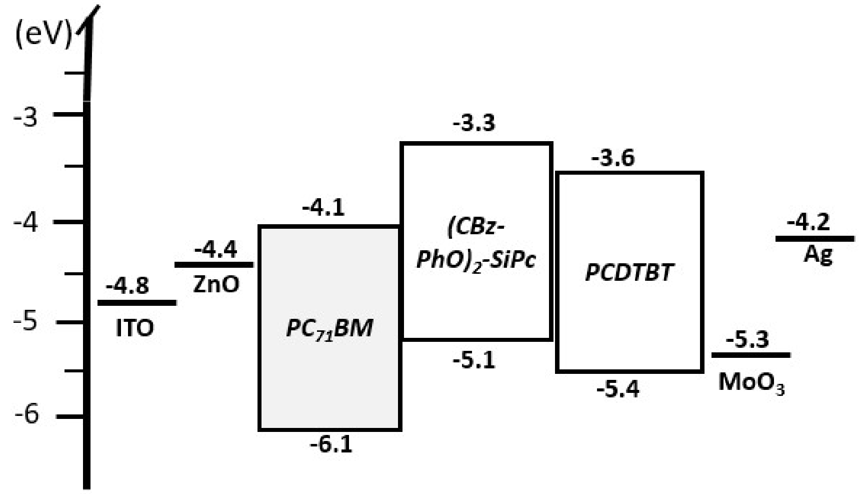

Figure S5. Energy level diagram of all materials used in ternary devices.

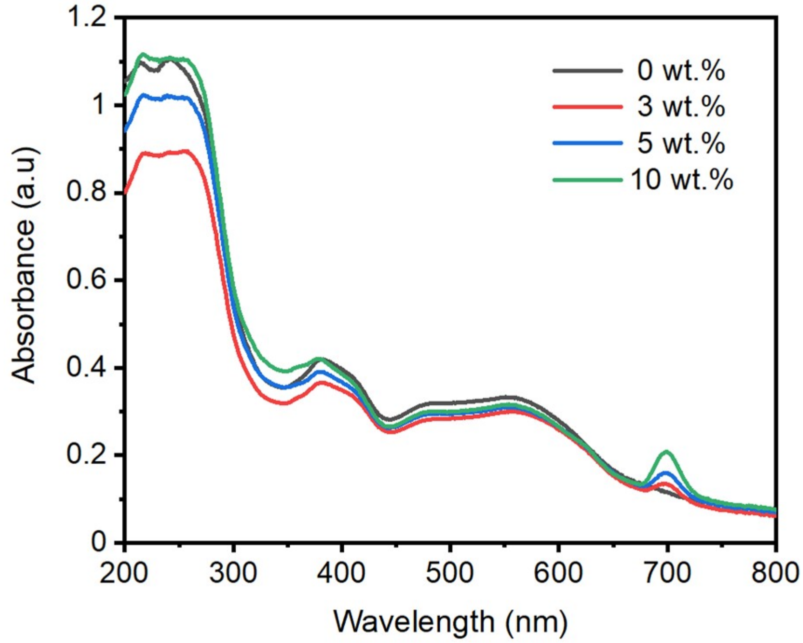

Figure S6. UV–vis absorption spectra of PCDTBT: $PC_{71}BM$ binary film and ternary films with different

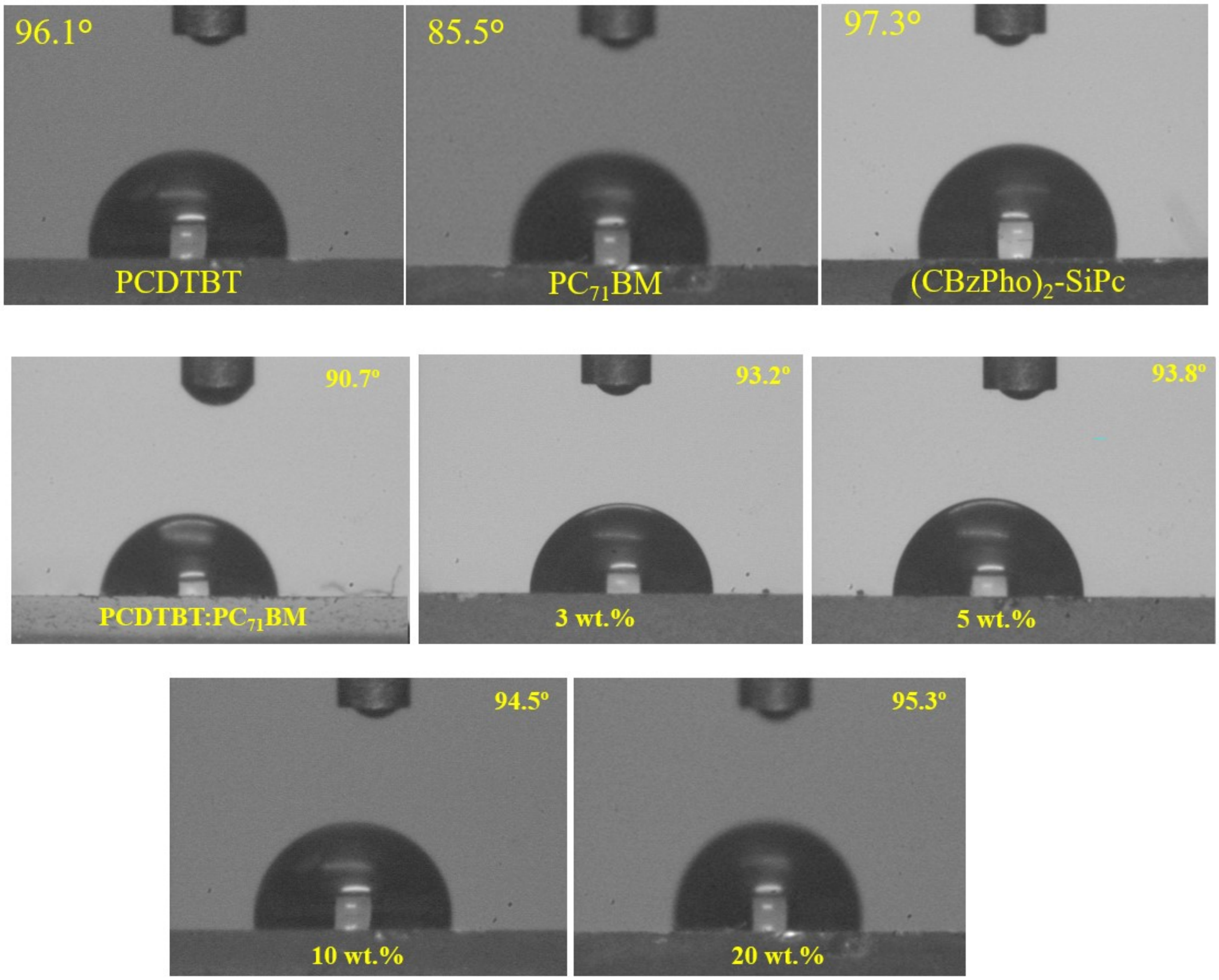

Figure S7: Water contact angle images of pristine films, binary and ternary films with different content of (CBzPho)2-SiPc varies from 0 to 20wt.%.

Table S1. Summary of H2O Contact Angle Measurements on ternary Films [(a)]

**Films** [(a)]	**Water Contact Angle (Deg)**	**Surface Energy (N mm^{-1})[(b)]**
PCDTBT:PC71BM	90.7 (± 0.6)	28.1 (± 0.7)
3 wt.% (CBzPho)2-SiPc	93.2 (± 0.4)	26.5 (± 0.7)
5 wt.% (CBzPho)2-SiPc	93.8 (± 0.7)	26.1 (± 0.5)
10 wt.% (CBzPho)2-SiPc	94.5 (± 0.6)	25.7 (± 0.4)
20 wt.% (CBzPho)2-SiPc	95.3 (± 0.5)	25.4 (± 0.3)

Table S2: Photovoltaic performance of ternary PCDTBT: $PC_{71}BM$ OPVs prepared by blade coating with various $(CBzPho)_2$-SiPc loadings.

$PCDTBT:PC_{71}BM:(CBzPho)_2$-SiPc	$(CBzPho)_2$-SiPc Wt. % [(a)]	Calibrated J_{SC} from EQE $(mA/cm^2)^b$	V_{OC} (V)	FF (%)	Calibrated PCE (%)
1:3:0	0	9.4 (±0.1)	0.842 (±0.001)	56.6 (±0.5)	4.5 (±0.1)
1:3:0.12	3	9.2 (±0.1)	0.835 (±0.001)	53.8 (±0.7)	4.1 (±0.1)
1:3:0.2	5	9.1 (±0.1)	0.842 (±0.001)	51.8 (±0.4)	3.9 (±0.1)
1.3:0.4	10	9.0 (±0.1)	0.845 (±0.002)	49.6 (±0.2)	3.8 (±0.1)
1:3:0.6	20	8.4 (± 0.3)	0.862 (±0.002)	48.6 (±0.2)	3.5 (±0.1)

All OPV devices were characterized under AM 1.5G, 100 $mWcm^{-2}$. Device areas is 1 cm^2. All values are average of more than 5 devices and the values in parentheses is the standard deviation. Blade coating process were performed in air. The base temperature for the blade process is 45 °C. [(a)] $(CBzPho)_2$-SiPc mass ratio in the blend with respect to total mass of PCDTBT: $PC_{71}BM$. [(b)] Current density (Jsc) calculated from integration wavelength of EQE curve.

Charge generation and recombination dynamics

In order to further investigate the exciton generation and dissociation process of the binary and ternary devices, the exciton generation rates *(G_{max})* and charge dissociation probabilities (P_{diss}) were analysed by characterization of photocurrent density (J_{ph}) against the effective voltage (V_{eff}), as shown in Figure S8. J_{ph} is defined as J_{ligh}–J_{dark}, where J_{Light} and J_{Dark} are the photocurrent densities under light illumination and in the dark, respectively. And $V_{eff} = V_0 - V_{appl}$, where V_0 is the voltage at which $J_{ph} = 0$, and V_{appl} is the applied bias.[1] The photogenerated excitons were almost dissociated into free charge carriers when V_{eff} reached <2 V, and thus J_{ph} could reach saturation (J_{sat}). We adopted the ratio of J_{ph}/J_{sat} to evaluate the exciton dissociation probability (P_{diss}) in the devices.[2] Assuming that all of the photogenerated excitons are dissociated and contributed to the current in the saturated regime due to sufficiently high electric field, the values of G_{max} can be obtained by $J_{sat} = qLG_{max}$, where q is the elementary charge and L is the thickness of the binary or ternary organic layer and Jsat is saturation current density at elevated V_{eff} (3.8V). Table S3 shows the values obtained for various (CBzPho)2-SiPc weight ratio loading. As expected the value of G_{max} for various addition of (CBzPho)2-SiPc are reduces compare to the baseline devices due to the high density of recombination sites as replicated in the performance of J-V curve. Under the short-circuit conditions, the (P_{diss}) values were 85.4%, 80.2%, 80.5%, and 81.5% for the devices with 0, 3, 5, and 10wt.% (CBzPho)2-SiPc, respectively.

The dynamics of recombination in PCDTBT:$PC_{71}BM$:(CBzPho)2-SiPc devices were investigated through the analysis of J_{SC} and V_{OC} under various light intensities (P_{light}). The J-V characterizations of binary and ternary devices with different content of $(CBzPho)_2$-SiPc as shown in the Figure S9. The plot ln (J_{SC}) versus ln (P_{light}) provides information on the degree of bimolecular recombination. The slope (α) implies close to unity means, negligible bimolecular recombination associated with the OPV devices.[3], [4] all the ternary devices slope (α) values

shows higher values than the baseline devices. (Figure S10a). In addition trap assisted monomolecular recombination extracted for the binary and ternary devices from the (Voc) versus Natural logarithmic (P_{light}) plot. Typically, a slope (S) equal to kT/q suggests the absence of bimolecular recombination, whereas an S ranging between 1 and 2 indicates the presence of monomolecular recombination and trap assisted recombination losses.[5], [6] It can be observed that the higher Slope (S) value of the PCDTBT: $PC_{71}BM$: (CBzPho)2-SiPc indicates (Figure S10b) that adding a small concentration of (CBzPho)2-SiPc results in a higher trap-assisted recombination associated, resulting in lower J_{SC} and FF as compared to those of the binary system.

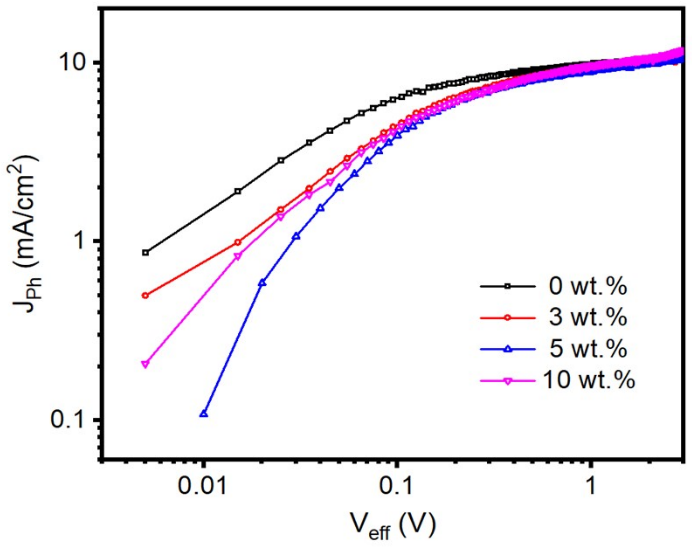

Figure S8. Photocurrent density *(J_{ph})* as a function of the effective voltage *(V_{eff})* of blade-coated PCDTBT:PC71BM:(CBzPho)2-SiPc devices with various (CBzPho)2-SiPc loadings.

Table S3: Charge generation parameters of blade coated PCDTBT:$PC_{71}BM$:$(CBzPho)_2$-SiPc ternary devices with different $(CBzPho)_2$-SiPc contents

$(CBzPho)_2$-SiPc Wt. %	J_{sat} (mA/cm^2)	G_{max} (cm^{-3}s^{-1})	P_{diss} (%)
0	11.8 (±0.1)	8.87 x 10^{21}	85.4
3	11.6 (±0.1)	8.72 x 10^{21}	80.2
5	12.5 (±0.2)	9.20 x 10^{21}	80.5
10	11.5 (± 0.1)	8.65 x 10^{21}	81.5

J_{sat} is the saturation current density, P_{diss} is the charge dissociation probability, G_{max} is the charge carrier generation.

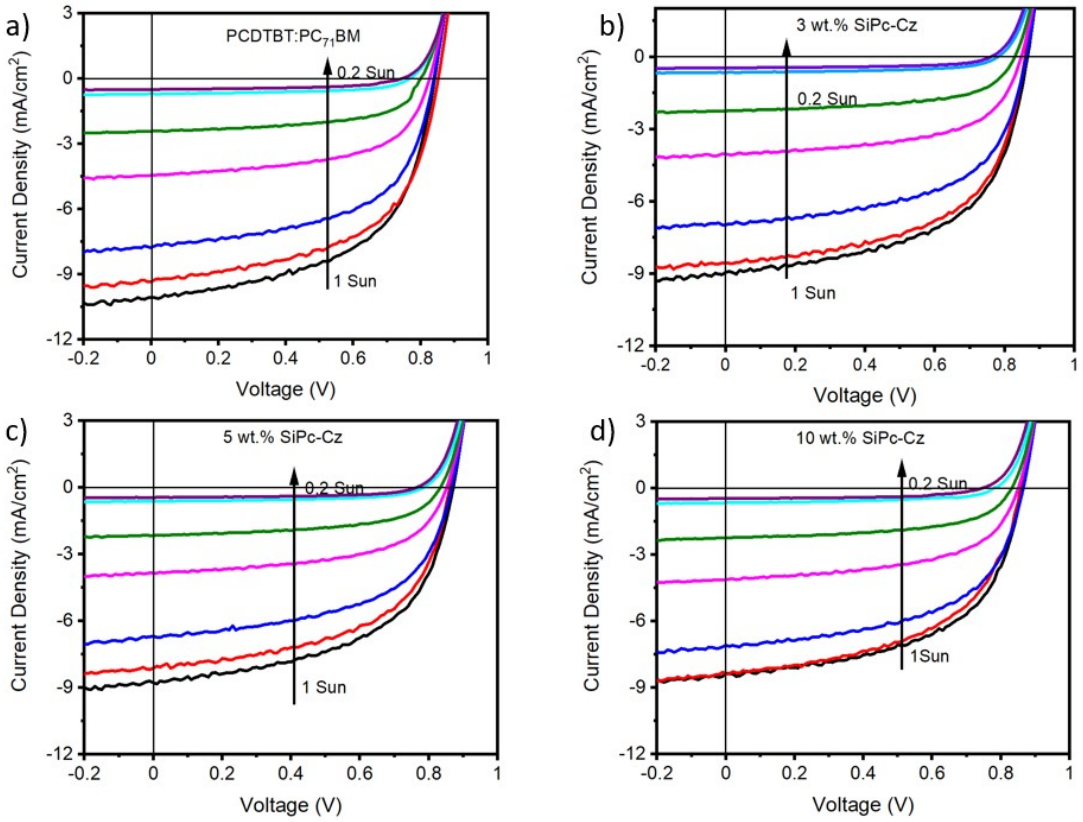

Figure S9. Light intensity dependence J-V curve of PCDTBT: $PC_{71}BM$ devices with various (CBzPho)2-SiPc loadings a) 0 wt.% b) 3 wt.% c) 5 wt.% and d) 10wt.% respectively. J-V curve measured under various light intensities ranging from 100 to 2 mW/cm^2.

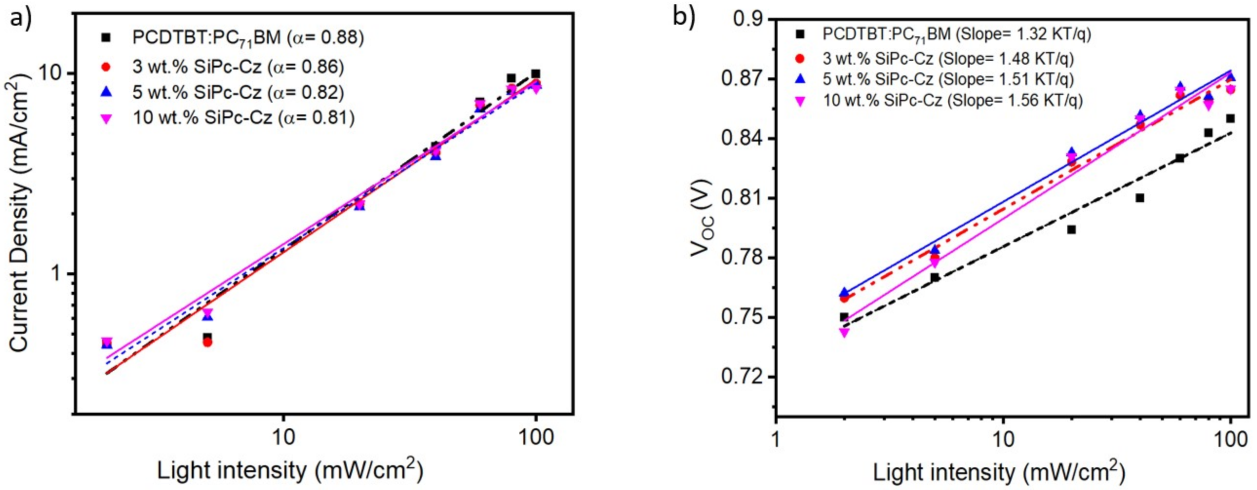

Figure S10. Light intensity dependence short circuit density and (a) open circuit voltage (b) of blade-coated PCDTBT: $PC_{71}BM$:$(CBzPho)_2$-SiPc devices with various $(CBzPho)_2$-SiPc loadings.

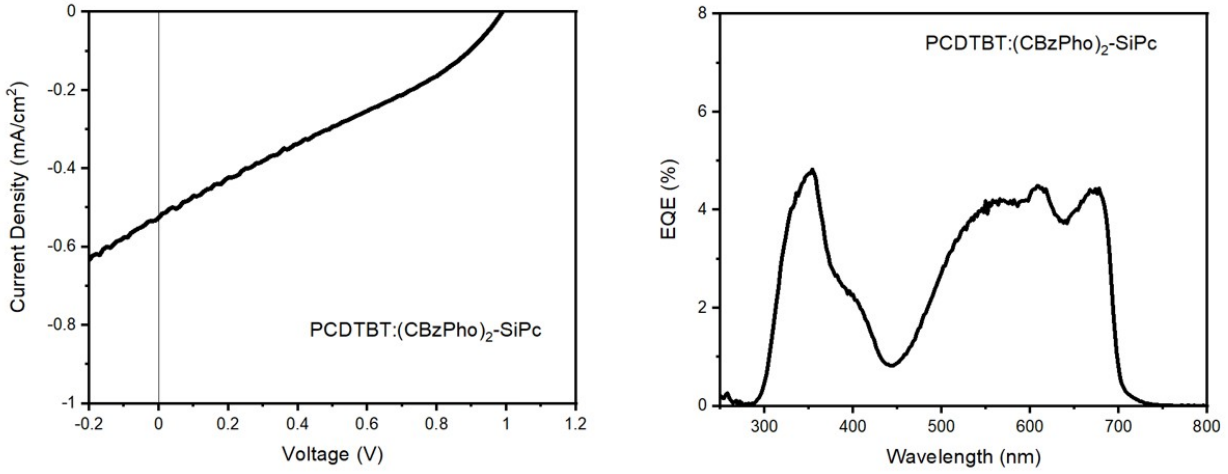

Figure S11. (a) J-V characteristics of PCDTBT: $CBzPho)_2$-SiPc Binary BHJ OPV devices fabricated by blade coating on ITO/PET substrates, (*V_{OC} 0.98 V, J_{SC} 0.56 mA/cm^2 FF 0.28 and PCE~ 0.15 %),* (b) corresponding EQE curves

Table S4. Energy levels of silicon phthalocyanine and Silicon Napththalocyanine derivatives incorporated into OPV devices

SiPc derivatives	HOMO (eV)	LUMO (eV)	Ref.
$(CBzPho)_2$-SiPc	-5.1	-3.3	This Work
$(HxN_3)_2$-SiPc	-5.4	-3.6	[7]
$(3XS)_2$-SiPc	-5.3	-3.4	[8][9]
X_2-SiPc	-5.3	-3.5	[10]
$(XF)_2$-SiPc	-5.9	-4.0	[11]
Carboxyl-SiPc	-5.4	-3.6	[12]
(Pys-SiPc)	-5.3	-3.6	[13]
(3XS-SiNc)	-4.8	-3.4	[14]

References:

[1] P. Bi and X. Hao, “Versatile Ternary Approach for Novel Organic Solar Cells: A Review,” *Sol. RRL*, vol. 3, no. 1, pp. 1–34, 2019, doi: 10.1002/solr.201800263.

[2] F. Paquin, J. Rivnay, A. Salleo, N. Stingelin, and C. Silva, “Multi-phase semicrystalline microstructures drive exciton dissociation in neat plastic semiconductors,” *J. Mater. Chem. C*, vol. 3, no. 207890, pp. 10715–10722, 2015, doi: 10.1039/b000000x.

[3] A. K. K. Kyaw *et al.*, “Intensity dependence of current-voltage characteristics and recombination in high-efficiency solution-processed small-molecule solar cells,” *ACS Nano*, vol. 7, no. 5, pp. 4569–4577, 2013, doi: 10.1021/nn401267s.

[4] D. Credgington, F. C. Jamieson, B. Walker, T. Q. Nguyen, and J. R. Durrant, “Quantification of geminate and non-geminate recombination losses within a solution-processed small-molecule bulk heterojunction solar cell,” *Adv. Mater.*, vol. 24, no. 16, pp. 2135–2141, 2012, doi: 10.1002/adma.201104738.

[5] L. Xiao *et al.*, “Multiple Roles of a Non-fullerene Acceptor Contribute Synergistically for High-Efficiency Ternary Organic Photovoltaics,” *Joule*, vol. 2, no. 10, pp. 2154–2166, 2018, doi: 10.1016/j.joule.2018.08.002.

[6] L. J. A. Koster, M. Kemerink, M. M. Wienk, K. Maturová, and R. A. J. Janssen, “Quantifying bimolecular recombination losses in organic bulk heterojunction solar cells,” *Adv. Mater.*, vol. 23, no. 14, pp. 1670–1674, 2011, doi: 10.1002/adma.201004311.

[7] T. M. Grant, T. Gorisse, O. Dautel, G. Wantz, and B. H. Lessard, “Multifunctional ternary additive in bulk heterojunction OPV: Increased device performance and stability,” *J. Mater. Chem. A*, vol. 5, no. 4, pp. 1581–1587, 2017, doi: 10.1039/c6ta08593h.

[8] M. T. Dang, T. M. Grant, H. Yan, D. S. Seferos, B. H. Lessard, and T. P. Bender, “Bis(tri-n-alkylsilyl oxide) silicon phthalocyanines: A start to establishing a structure property relationship as both ternary additives and non-fullerene electron acceptors in bulk heterojunction organic photovoltaic devices,” *J. Mater. Chem. A*, vol. 5, no. 24, pp. 12168–12182, 2017, doi: 10.1039/c6ta10739g.

[9] S. Honda, S. Yokoya, H. Ohkita, H. Benten, and S. Ito, “Light-harvesting mechanism in polymer/fullerene/dye ternary blends studied by transient absorption spectroscopy,” *J. Phys. Chem. C*, vol. 115, no. 22, pp. 11306–11317, 2011, doi: 10.1021/jp201742v.

[10] T. M. Grant and J. L. Brusso, “Bis(trialkylsilyl oxide) Silicon Phthalocyanines: Understanding the Role of Solubility in Device Performance as Ternary Additives in

Organic Photovoltaics´," 2020, doi: 10.1021/acs.langmuir.9b03772.

[11] T. M. Grant, R. White, E. Thibau, Z. Lu, and T. P. Bender, "Donor / Acceptor Properties of Fluorophenoxy Silicon Phthalocyanines within Electronic Supporting Information .," 2015.

[12] E. Zysman-Colman *et al.*, "Solution-Processable Silicon Phthalocyanines in Electroluminescent and Photovoltaic Devices," *ACS Appl. Mater. Interfaces*, vol. 8, no. 14, pp. 9247–9253, 2016, doi: 10.1021/acsami.5b12408.

[13] L. Ke *et al.*, "A Series of Pyrene-Substituted Silicon Phthalocyanines as Near-IR Sensitizers in Organic Ternary Solar Cells," *Adv. Energy Mater.*, vol. 6, no. 7, pp. 1–13, 2016, doi: 10.1002/aenm.201502355.

[14] B. Lim, J. T. Bloking, A. Ponec, M. D. McGehee, and A. Sellinger, "Ternary bulk heterojunction solar cells: Addition of soluble NIR dyes for photocurrent generation beyond 800 nm," *ACS Appl. Mater. Interfaces*, vol. 6, no. 9, pp. 6905–6913, 2014, doi: 10.1021/am5007172.
